# The context of illicit drug overdose deaths in British Columbia, 2006

**DOI:** 10.1186/1477-7517-6-9

**Published:** 2009-05-29

**Authors:** Jane A Buxton, Trevor Skutezky, Andrew W Tu, Bilal Waheed, Alex Wallace, Sunny Mak

**Affiliations:** 1 British Columbia Centre for Disease Control, Vancouver, British Columbia, Canada; 2 University of British Columbia, School of Population and Public Health, Vancouver, British Columbia, Canada; 3 British Columbia Coroner's Service, Burnaby, British Columbia, Canada

## Abstract

**Background:**

Illicit drug overdose deaths (IDD) relate to individual drug dose and context of use, including use with other drugs and alcohol. IDD peaked in British Columbia (BC) in 1998 with 417 deaths, and continues to be a public health problem. The objective of this study was to examine IDD in 2006 in BC by place of residence, injury and death, decedents' age and sex and substances identified.

**Methods:**

IDD data was obtained through the BC Coroners Office and entered into SPSS (version 14). Fisher's exact and Pearson's χ^2 ^were used for categorical data; Mann-Whitney U-test for continuous variables. Rates were calculated using 2006 population estimates.

**Results:**

We identified 223 IDD in BC; 54 (24%) occurred in Vancouver. Vancouver decedents (compared to those occurring outside Vancouver) were older (mean age 43.9 vs. 39.2 years; p < 0.01) and more likely to be male (90.7% vs. 77.5%; p = 0.03). Provincially Aboriginal ethnicity was reported for 19 deaths; 13 (30.2%) of 43 females and 6 (3.3%) of 180 males (p = < 0.001).

Cocaine was identified in 80.3%, opiates 59.6%, methadone 13.9%, methamphetamine/amphetamine 6.3%, and alcohol in 22.9% of deaths. Poly-substance use was common, 2 substances were identified in 43.8% and 3 or more in 34.5% of deaths. Opiates were more frequently identified in Vancouver compared to outside Vancouver (74.1% vs. 55.0%) p = 0.015.

**Conclusion:**

Collaboration with the Coroner's office allowed us to analyze IDD in detail including place of death; cocaine, opiates and poly-substance use were commonly identified. Poly-substance use should be explored further to inform public health interventions.

## Background

Illicit drug overdose deaths (IDDs) are a significant public health problem in British Columbia (BC). They peaked in 1998 with 417 deaths, of which 46% were Vancouver residents; in 2005, 218 deaths were reported (personal communication, BC Coroner's office, September 2008). Delivery of effective and responsive public health interventions to combat IDDs relies on ongoing observation of the changing landscape of drug use patterns.

Current literature suggests a trend of increasing poly-substance use by illicit drug users. The concomitant use of multiple substances emerged as a key risk factor in illicit drug overdoses in New York City between 1990 and 1998; with heroin, cocaine and alcohol being the most common drug combinations.[1] It has been suggested that tracking single drug usage is insufficient to guide public health interventions.[1] More recent studies reinforce these findings; between 1990 and 2005 in New Mexico, USA, 47.2% of all unintentional drug overdoses were caused by the presence of two or more substances.[2]

The Downtown East Side of Vancouver (DTES) is considered to be the centre of the injection drug use epidemic in Vancouver.[3] Previously, IDDs in BC were classified by township of residence of the decedent;[4] place of death and deaths occurring in non-BC residents were not reported.

The objective of this study was to determine (i) the demographic (age, sex and ethnicity) and geographic (place of injury, death and residence) distribution and (ii) the role of poly-substance use, of BC 2006 IDDs.

## Methods

Ethics approval was received from University of British Columbia Behavioural Research Ethics Board (H08-00333). Sex, ethnicity (Aboriginal or non-Aboriginal as reported by family and associates of the decedent), age, geographic details (township of residence, injury and death), toxicological results and recorded cause of death were requested from the BC Coroners Office for all cases coded as IDD in BC for the 2006 calendar year.

We compared the township of injury, death and residence at time of death, to determine the most appropriate for mapping purposes. We requested six-digit postal codes of cases that were residents of Vancouver and converted these to one of 6 Vancouver Local Health Areas (LHA). To maintain confidentiality the Vancouver postal code file was not linked to other demographic data. The IDD rates per 100,000 were mapped using ArcGIS 9.2 (ESRI Inc., Redlands, CA) by LHA using city for the province of BC and by LHA of residence within Vancouver.

### Toxicology

The BC Coroners Office conducts a toxicologic examination for all deaths where the abuse of street drugs is suspected. The decedent is screened for alcohol, cocaine, morphine, amphetamines, cannabinoids and methadone. A prescription drug-screen tests for prescription and over-the-counter medication in addition to methadone and methamphetamine. Lysergic Acid Diethylamide (LSD) and phenylcyclohexylpiperidine, (PCP) are only screened on request.[3]

Blood and urine are usually provided for cocaine, benzoylecgonine (a metabolite of cocaine), alcohol, morphine, 6-monoacetylmorphine (6-MAM, a metabolite of heroin), acetaminophen, methadone, codeine, amphetamines, gamma-hydroxybutanate (GHB) and ecstasy concentrations. (Personal communication Bilal Waheed, BC Coroners Service, June 20, 2008) We could not determine if methadone was prescribed or illegally obtained therefore we reported methadone separately. We categorized cocaine and benzoylecgonine as cocaine; heroin, morphine, 6-MAM and codeine were categorized as opiates (excluding methadone).

The median blood concentration was compared with the average lethal limit for each substance. Where more than two days was reported between death and autopsy, blood samples were generally not taken. Therefore cases in which postmortem metabolism (altering the toxicological findings) may have occurred were excluded from quantitative comparison of blood levels.

### Data Analysis

Data was received in Excel format and inputted into SPSS (version 14.0 for windows SPSS Inc., Chicago, Illinois, USA). Descriptive data was compared using Fisher's exact test for 2 × 2 categorical data, Pearson's χ^2 ^for m × n categorical data, and Mann-Whitney U-test for continuous variables. A level of significance of α = 0.05 was used. Substance levels were converted to standard units to allow for comparison and statistical analysis. Rates were age-adjusted using the direct method and the 2006 BC population from P.E.O.P.L.E. 32 as the standard.[5]

## Results

The Coroners Office provided data for 225 cases. One case was classified as death due to a medical condition with illicit drugs as a contributing factor; another was classified as leukoencephalopathy (a condition affecting the brain associated with smoking heroin but not an acute overdose death). Both cases were removed from the analysis. Thus we investigated 223 cases, of these five were deemed suicide; 43 cases (19.3%) were female. Nineteen cases (8.4%) were reported as Aboriginal, 13 (30.2%) of females and 6 (3.3%) of males (p = < 0.001). Mean age at death was 40.3 years (range 17.4 to 66.8 years).

Townships of injury and of death were available for all 223 cases and were identical. Township of residence was missing in 13 cases; 6 decedents were residents of Alberta; (3 of these died in the Interior of BC, one on Vancouver Island and 2 in the lower mainland), see table 1. To present the most complete data, township of injury was used to map IDDs province wide. Fifty-four (24.2%) of 2006 BC deaths occurred in Vancouver. Deaths occurring in Vancouver were more likely to be male and older than those occurring outside Vancouver, see table 2.

**Table 1:** Comparison of place or injury/death and residence of 2006 IDD by health authority

Health Authority	Place of injury/death	Residence
Interior	38	37
Fraser	81	77
Vancouver Coastal	63	53
Vancouver Island	34	32
Northern	7	5
Missing	0	13

BC	223	217 (6 Alberta)

**Table 2:** Demographic of 2006 IDD deaths in British Columbia by place of injury/death

	Vancouver (n = 54)	Outside Vancouver (n = 169)	p-value
Mean age at death (SD)	43.9 (9.9)	39.2 (11.3)	< 0.01
Gender (%)			0.03
Male	49 (90.7)	131 (77.5)	-
Female	5 (9.3)	38 (22.5)	-
Ethnicity (%)*			n.s.
Aboriginal	4 (7.4)	15 (8.9)	-
Non-Aboriginal	50 (92.6)	154 (91.1)	-
Death Premise (%)			0.05
Residential	46 (85.2)	111 (65.7)	-
Medical facility	6 (11.1)	37 (21.9)	-
Transport area	1 (1.9)	9 (5.3)	-
Other**	1 (1.9)	12 (7.1)	-

*Aboriginal ethnicity was as reported to Coroner by associates, family etc. and not necessarily accurate.

** Note that other includes: other specified place (5), wooded area (2), public building (1), correctional institution (2), detoxification centre (1) unknown (1), null (1).

**Figure 1:**
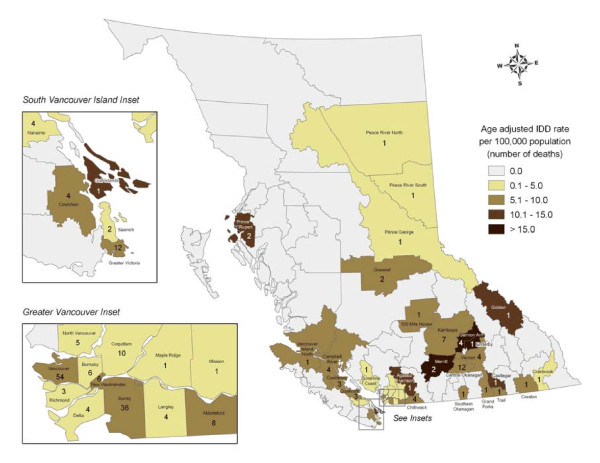
**Illicit Drug Deaths in British Columbia by local health area, 2006 (n = 223)**. Illicit Drug Deaths (IDD) are mapped by place of injury. Age-adjusted rates of IDD in rural local health areas with small number of IDD should be interpreted with caution due to unstable rates.

**Figure 2:**
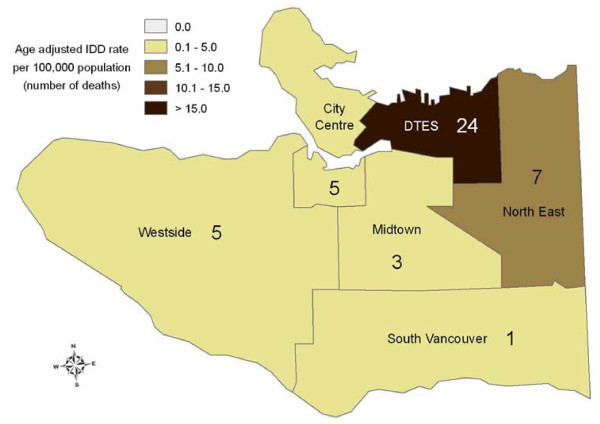
**Illicit Drug Deaths in the City of Vancouver by local health area, 2006**. Illicit Drug Deaths (IDD)are mapped by place of residence. Three additional IDD could not be mapped due to missing geolocator information.

Age-adjusted IDD rates per 100,000 by LHA of injury/death for BC are shown in figure 1. Of the 54 deaths that occurred within Vancouver, 45 were Vancouver residents, 4 resided in the lower mainland, 1 in BC interior, 1 out of province, and 3 had no residency information. The six-digit postal code of residence was provided for 45 of the 48 cases in which Vancouver was identified as township of residence, (3 were missing). Twenty-four (53%) of Vancouver resident cases where postal code was known lived in Local Health Area 162, which includes the DTES of Vancouver, see figure 2.

Toxicology results are detailed in table 3. Cocaine was identified in 179 (80.3%) deaths; 44 (81.5%) of Vancouver cases and 135 (79.9%) cases outside Vancouver. Opiates (not including methadone) were found in 133 (59.6%) deaths with 6-MAM identified in 49 cases. Opiates were identified more frequently in Vancouver (40; 74.1%) vs. outside Vancouver (93; 55.0%) (p = 0.016). Median blood morphine level was 0.17 mg/L (range 0.01–1.40 mg/L); 26.3% of cases were above the average lethal limit of 0.32 mg/L.[6] Alcohol was identified in 51 (22.9%) deaths; no cases were above the lethal alcohol limit.[7]

**Table 3:** Drugs identified in toxicological screens of illicit drugs.

Drugs (%)	Vancouver (n = 54)	Outside Vancouver (n = 169)	Total (n = 223)
Opiates* alone	2 (3.7)	5 (3.0)	7 (3.1)
Opiates and cocaine alone	17 (31.5)	32 (18.9)	49 (22.0)
Opiates and alcohol alone	2 (3.7)	4 (2.4)	6 (2.7)
Opiates and other drug(s)^†^	3 (5.6)	11 (6.5)	14 (6.3)
Opiates, cocaine, and other drug(s)	5 (9.3)	28 (16.6)	33 (14.8)
Opiates, alcohol, and other drug(s)	0	2 (1.2)	2 (0.9)
Opiates, cocaine, and alcohol alone	9 (16.7)	9 (5.3)	18 (8.1)
Opiates, cocaine, alcohol, and other drug(s)	2 (3.7)	2 (1.2)	4 (1.8)
Cocaine alone	4 (7.4)	30 (17.8)	34 (15.2)
Cocaine and alcohol alone	0	10 (5.9)	10 (4.5)
Cocaine and other drug(s)	6 (11.1)	20 (11.8)	26 (11.7)
Cocaine, alcohol, and other drug(s)	1 (1.9)	4 (2.4)	5 (2.2)
Methadone alone	0	1 (0.6)	1 (0.4)
Other**	3 (5.6)	11 (6.5)	14 (6.3)

*Opiates include heroin, morphine, and codeine and exclude methadone. ^†^Other drugs include methamphetamine, amphetamine, benzodiazepines, and anti-depressants. Methadone was considered 'other drug' in cases where it was not present alone.

**Alcohol was identified in 6 cases

Both morphine and cocaine were detected in 99 (44.4%) cases (55.3% of cocaine positive cases). Alcohol was detected in 30 (22.6%) opiate positive cases; no significant difference was observed in the median blood morphine level between cases where alcohol was or was not detected. Morphine was present in 28 (54.9%) of alcohol positive cases.

Methadone was identified in 31 (13.9%) deaths, of which 7 (22.6%) occurred in Vancouver. Of the 31-methadone positive cases, cocaine was present in 18 (58.1%) cases and opiates were present in 7 (22.6%) cases, see table 4. Median blood methadone level was 0.43 mg/L (range 0.10–4.10 mg/L); 20.0% of cases were above the average lethal limit of 1 mg/L.[6] Methamphetamine/amphetamine was present in 14 (6.3%) cases, of which 9 were also cocaine positive and 8 positive for opiates.

**Table 4:** Toxicological findings of methadone positive cases.

Drugs	Number of Cases n (%)
Methadone alone	1 (3.2)
Methadone and opiate present	4 (12.9)
Methadone and cocaine present	15 (48.4)
Methadone, cocaine, and opiates present	3 (9.7)
Methadone and other combination	8 (25.8)
Total methadone	31

Poly-substance use was common, and included other illicit drugs, prescription drugs and alcohol. Two substances were identified in 43.8% and 3 or more in 34.5% of deaths (see table 5). Antidepressants and benzodiazepines were present in 10.3% and 3.6% of deaths respectively. A medical cause of death and/or other factors contributing to death fields were populated in 64 cases. Of note were the eight acute myocardial infarction deaths (mean age 37.1 years), all of which were associated with cocaine.

**Table 5:** Poly-substance use in BC, n (%).

	One Substance	Two Substances	Three or more Substances	Total
Vancouver	7 (13.0%)	26 (48.1%)	21 (38.9%)	54
Outside Vancouver	39 (23.1%)	74 (43.8%)	56 (33.1%)	169
Total	46	100	77	223

## Discussion

Working collaboratively with the Coroner's Office enabled us to analyse IDD data in detail and identify demographic differences. A significantly higher proportion of Vancouver IDD were male; and significantly more females were reported as Aboriginal than their male counterparts. Other studies have identified Aboriginal females to have high-risk drug using behaviours, therefore interventions should be gender and culturally appropriate.[8] The mean age at death was 40.3 years; which suggests that the decedents were not necessarily young or inexperienced users.

The current study illustrates geographic variations. Although DTES has a high IDD rate, nearly half of the deaths occurring in Vancouver were outside the DTES and three-quarters of all BC deaths occur outside Vancouver; supporting the need for accessible and acceptable mental health and addiction services to be available throughout BC. To allow the most complete data to be mapped we used city of injury/death and for more precise details within Vancouver, we used postal code of residence; therefore these data are not comparable. In the future, global positioning systems will enable the coroner to record place of death more precisely.

Coroner's case reports of IDD occurring in 1997–99 were previously reviewed; in 2006 compared to 1997–99, cocaine was more prevalent (>80% vs. 50%) and opiates less prevalent (60% vs. 74%).[9] Our finding of the predominance of cocaine compared to opiates, differs from other cities. In Sydney, Australia, heroin was reported in 90% of forensic deaths,[10] and opiates continue to be the leading cause of IDDs in New Mexico.[2] However, we do not know if drug use in BC reflects drug of choice or availability of substances. Our observed trend may be a response of the local drug market to the external global heroin supply as explored by Wood *et al*.[11]

We found poly-substance use was common; a single substance was identified in <20% of IDD in 2006. This is consistent with other studies conducted in Vancouver and in North America.[1,12] However we do not know if the substances were used simultaneously or sequentially, the route of substance administration nor if each used for specific effects.

Alcohol prevalence increased from 1997–99 to 2006 (17% vs. 22.9%) despite a Vancouver Police Department policy introduced in1999 to remove rice wine from 'corner' stores in order to reduce a source of inexpensive alcohol in DTES.[13] However, other cheap non-beverage alcohol sources such as alcohol containing mouthwash continue to be readily available. Co-administration of alcohol can substantially increase the likelihood of a fatal outcome following injection of heroin, due to the potentiation of the respiratory depressant effects of heroin.[14] Research has suggested a negative correlation between blood morphine and blood alcohol levels in decedents.[15] In our study alcohol was detected in less than a quarter of cases where opiates were identified; by comparison, Darke *et al *reported that 41.1% of heroin overdose deaths in Sydney, Australia were alcohol positive.[16] We found no significant difference in the blood morphine levels of cases where alcohol was present in conjunction with morphine compared to those cases where it was not. We found the majority of blood morphine concentrations well below the lethal limit, supporting the suggestion that morphine concentrations per se are not adequate to attribute cause of overdose.[16]

The circumstances surrounding the deaths and context of drug use are unknown; Binswanger found drug overdose was the leading cause of death among former prison inmates immediately after release.[17] We found the majority of deaths occurred in residential settings; however it is uncertain if the decedent was alone at the time of drug use. Users should be encouraged to adopt safer practices including using Vancouver's medically supervised injection facility (Insite), or the 'buddy system' so in the event of an overdose the 'buddy' can call for help. Pilot projects in several US jurisdictions, have provided users with naloxone and report positive results.[18]

No IDDs have occurred in Vancouver's medically supervised injection facility (Insite) since it opened in March 2003.[19] A recent study estimated of the 453 overdoses occurring at Insite, between 8 and 51 deaths were averted if these had occurred outside the facility.[20] However, the effect on overall IDD is unknown. Persons may use Insite for a small proportion of their injections,[19] and are more likely to report injecting heroin than cocaine.[21]

There are several limitations to this study that should be considered. With the use of the place of injury variable to calculate rates instead of place of residence, rates must be interpreted with caution. These rates may be influenced by the location of medical facilities or by the mobility of this population. However, because of the mobility of this population, the place of residence variable, which describes the last known residence of the decedents, may not accurately represent the decedent's residence at time of death.[22] Also, there was only an 8% discordance between place of injury and place of residence. Many of the 83 BC LHAs have no or few IDDs in one-year, therefore rates may be unstable. This limitation may be mitigated by using multiple years of data or larger geographic aggregations. However reporting IDD by the 16 BC Health Service Delivery Areas loses specificity in the ability to see smaller scale spatial patterns. Toxicological substance concentrations must be interpreted with caution as they may be confounded by a number of factors. Each individual case presents a unique combination of substances, routes of administration, underlying health problems, time of last dose prior to death and level of tolerance. Toxicology at autopsy may not represent the situation at time of death, variation in the time elapsed between and anatomical location of samples may affect substance concentration at postmortem.

Collaboration with the Coroner's office allowed us to analyze IDD in detail including place of death and drugs identified. We found that cocaine, opiates and poly-substance use were common. Public health interventions should address and further explore poly-substance use and not focus on individual substance use alone.

## Competing interests

The authors declare that they have no competing interests.

## Authors' contributions

JB contributed to the conception and design of the paper. TS conducted the statistical analysis and drafted the manuscript. AWT contributed to the data management and statistical analysis of the paper. BW and AW provided the data for the study. SM contributed to the GIS mapping of the data. All authors contributed to and approved the final manuscript.
